# Glycogenin is Dispensable for Glycogen Synthesis in Human Muscle, and Glycogenin Deficiency Causes Polyglucosan Storage

**DOI:** 10.1210/clinem/dgz075

**Published:** 2019-10-19

**Authors:** Kittichate Visuttijai, Carola Hedberg-Oldfors, Christer Thomsen, Emma Glamuzina, Cornelia Kornblum, Giorgio Tasca, Aurelio Hernandez-Lain, Joakim Sandstedt, Göran Dellgren, Peter Roach, Anders Oldfors

**Affiliations:** 1 Department of Pathology and Genetics, Sahlgrenska Academy, University of Gothenburg, Gothenburg, Sweden; 2 National Metabolic Service, Starship Children’s Hospital, Auckland, New Zealand; 3 Department of Neurology, Bonn University Hospital, Bonn, Germany; 4 Unità Operativa Complessa di Neurologia, Dipartimento di Scienze dell’Invecchiamento, Neurologiche, Ortopediche e della Testa-Collo, Fondazione Policlinico Universitario A. Gemelli IRCCS, Rome, Italy; 5 Department of Pathology and Research Institute, Hospital 12 de Octubre, Madrid, Spain; 6 Department of Clinical Chemistry, Institute of Biomedicine, University of Gothenburg, Gothenburg, Sweden; 7 Department of Cardiothoracic Surgery, Sahlgrenska University Hospital, Gothenburg, Sweden; 8 Department of Biochemistry and Molecular Biology, Indiana University School of Medicine, Indianapolis, Indiana

## Abstract

**Context:**

Glycogenin is considered to be an essential primer for glycogen biosynthesis. Nevertheless, patients with glycogenin-1 deficiency due to biallelic *GYG1* (NM_004130.3) mutations can store glycogen in muscle. Glycogenin-2 has been suggested as an alternative primer for glycogen synthesis in patients with glycogenin-1 deficiency.

**Objective:**

The objective of this article is to investigate the importance of glycogenin-1 and glycogenin-2 for glycogen synthesis in skeletal and cardiac muscle.

**Design, Setting, and Patients:**

Glycogenin-1 and glycogenin-2 expression was analyzed by Western blot, mass spectrometry, and immunohistochemistry in liver, heart, and skeletal muscle from controls and in skeletal and cardiac muscle from patients with glycogenin-1 deficiency.

**Results:**

Glycogenin-1 and glycogenin-2 both were found to be expressed in the liver, but only glycogenin-1 was identified in heart and skeletal muscle from controls. In patients with truncating *GYG1* mutations, neither glycogenin-1 nor glycogenin-2 was expressed in skeletal muscle. However, nonfunctional glycogenin-1 but not glycogenin-2 was identified in cardiac muscle from patients with cardiomyopathy due to *GYG1* missense mutations. By immunohistochemistry, the mutated glycogenin-1 colocalized with the storage of glycogen and polyglucosan in cardiomyocytes.

**Conclusions:**

Glycogen can be synthesized in the absence of glycogenin, and glycogenin-1 deficiency is not compensated for by upregulation of functional glycogenin-2. Absence of glycogenin-1 leads to the focal accumulation of glycogen and polyglucosan in skeletal muscle fibers. Expression of mutated glycogenin-1 in the heart is deleterious, and it leads to storage of abnormal glycogen and cardiomyopathy.

Glycogen is a large, branched polysaccharide that is present in all tissues but mainly in the liver, skeletal muscle, and heart, and is a readily available source of energy. In the liver, glycogen is used to keep blood glucose at physiological levels, whereas in muscle glycogen is used as an energy source for muscle cells. Formation of glycogen (glycogenesis) from glucose is a multistep process regulated by various enzymes (1, 2). The enzyme glycogenin is considered to be essential for initiating de novo glycogen synthesis. By autoglucosylation, glycogenin generates an oligosaccharide chain of approximately 7 to 12 glucose residues, which are linearly linked by α1,4-glycosidic bonds and covalently linked to the glycogenin apoprotein by a tyrosine-O-glucose binding. Glycogen synthase adds further glucose molecules to the priming oligosaccharide chain by formation of more α1,4-glycosidic linkages. Branching enzyme adds glucose molecules by α1,6-glycosidic linkages, which leads to branching of the molecule. By this process, the glycogen molecule grows to form the glycogen β particle, consisting of approximately 30 000 glucose molecules, and these β particles can be linked together to form even larger α particles. Glycogenin is found in 2 isoforms, glycogenin-1 and glycogenin-2, which are encoded by 2 separate genes, *GYG1* and *GYG2*, respectively. Glycogenin-1 is a 39-kDa protein (the GN1L-isoform) that is ubiquitously expressed. Functional glycogenin-2, a 55-kDa protein (the α-isoform) that resembles glycogenin-1 structurally and functionally, is also expressed in the liver (3, 4). In glycogenin-1, the tyrosine-O-glucose bond is at position Tyr195, whereas in glycogenin-2 the glucose is attached to Tyr228.

Glycogen storage disease type XV/polyglucosan body myopathy 2 (OMIM#613507/#616199) is caused by biallelic mutations in the *GYG1* gene. Since the first report in 2010 (5), more than 30 patients with glycogenin-1 deficiency have been described. Most of these patients had adult-onset, slowly progressive myopathy and muscle weakness without cardiomyopathy (6–14), but there have also been reports of patients presenting with cardiomyopathy without muscle weakness, leading to severe cardiac failure (5, 15). Patients with *GYG1* mutations are characterized by either the absence of glycogenin-1 or the expression of nonfunctional glycogenin-1, and there is storage of glycogen and polyglucosan in the affected tissues.

Despite the fact that glycogenin is considered to be essential for de novo glycogen synthesis (16), glycogen is present in the skeletal muscle of glycogenin-1–deficient patients. This finding challenges the generally accepted concept that glycogenin is required for glycogen synthesis, and it has been speculated that glycogenin-2 may act as an alternative primer for glycogen synthesis (5). In 1 study, Western blot analysis of glycogenin-2 was performed on muscle from 2 patients with glycogenin-1 deficiency, and bands believed to be glycogenin-2 were identified in the patients, but no functional glycogenin-2 was demonstrated (12).

To further investigate the hypothesis that upregulation of expression of functional glycogenin-2 may substitute for glycogenin-1 deficiency in cardiac and skeletal muscle, we investigated the expression of glycogenin-1 and glycogenin-2 by immunohistochemistry and Western blot analysis in liver, heart, and skeletal muscle from controls and in heart and skeletal muscle from patients with biallelic *GYG1* mutations.

## Methods

### Participants

This study included biopsy specimens from 5 previously described unrelated patients with biallelic pathogenic *GYG1* mutations (5–7, 15). A summary of the results of clinical and laboratory examinations is given in Table 1. Patients 1, 2, and 3 (Pt1, Pt2, and Pt3) had pure myopathy without signs or symptoms of cardiomyopathy, whereas patients 4 and 5 (Pt4 and Pt5) presented with cardiomyopathy and minor or no signs and symptoms of skeletal myopathy. Skeletal muscle specimens from Pt1, Pt2, and Pt3 were obtained by open biopsy. Cardiac muscle specimens were obtained by endomyocardial biopsy and heart explants after transplantation in Pt4 and Pt5. Control skeletal muscle (M1, M2, and M3) included muscle biopsy specimens from individuals who had been investigated for a possible muscle disorder but in whom the investigation excluded muscle disease. There were control cardiac muscles from 2 individuals with no apparent cardiac disease who had donated their hearts for transplantation, but who had been excluded (H1 and H2). Two additional cardiac muscle controls (H3 and H4) were from explanted hearts in patients with dilated cardiomyopathy. Control liver came from liver biopsies performed for diagnostic purposes. In 2 cases, liver disease was excluded after investigation (L1 and L2), and 2 cases showed fibrosis (L3 and L4). The liver and muscle specimens used as immunohistochemical staining controls were obtained from routine diagnostic work at Sahlgrenska University Hospital, Gothenburg, Sweden.

**Table 1.** Summary of patients with glycogenin-1 deficiency who were investigated in this study.

Patient	Pt1	Pt2	Pt3	Pt4	Pt5
Sex	M	F	M	M	M
Origin	Italy	Spain	Germany	New Zealand and UK	Sweden
Age at onset, y	82	65	62	34	Probably childhood
Initial symptoms	Progressive pain and weakness in upper left arm	Difficulties climbing stairs and walking	Difficulties climbing stairs and walking	Shortness of breath, chest pain on exertion, lethargy, and palpitations	Shortness of breath and upper arm weakness
Age at examination, y	84	72	72	48	27
Clinical features	Weakness in proximal and distal upper and lower limbs, partly asymmetric	Shoulder and hip girdle muscle weakness	Hip girdle and thigh muscle weakness and atrophy; milder asymmetric weakness of shoulder girdle and upper arm muscles	Cardiac failure. Heart transplantation at age 48	Cardiac failure with reduced ejection fraction. Cardiac arrhythmias. Minor weakness in upper arms, neck flexion, and foot dorsiflexion
Cardiomyopathy	No	No	No	Yes	Yes
Muscle histopathology	Myopathy with α-amylase resistant,PAS-positive inclusions and nemaline rods. Increase in interstitial fibrous and fat tissue	Partially α-amylase resistant,PAS-positive inclusions	Partially α-amylase resistant, PAS-positive inclusions. Moderate increase in interstitial fibrous and fat tissue	Variability in fiber size and rare fibers with partially α-amylase resistant, PAS-positive inclusions	Smaller muscle fiber size than normal; glycogen deficiency
Heart histopathology	Not performed	Not performed	Not performed	PAS-positive inclusions in almost all cardiomyocytes, with partial removal by α-amylase	PAS-positive inclusions in almost all cardiomyocytes, with partial removal by α-amylase
DNA analysis	Homozygous c.2T > A	Homozygous c.46G > C	Compound heterozygous c.7G > C c.143 + 3G > C	Homozygous c.304G > C	Compound heterozygous c.248C > T c.487delG
Predicted protein change	Start codon mutation	p.Ala16Pro	p.Asp3Glufs*4 p.spl	p.Asp102His	p.Thr83Met p.Asp163Thrfs*5
Previous report in which patient was described	(7)	Patient P4 (6)	Patient P5 (6)	(15)	(5)
Tissue investigated in this study	Skeletal muscle	Skeletal muscle	Skeletal muscle	Endomyocardial biopsy and heart explant	Endomyocardial biopsy

Abbreviations: F, female; M, male; *GYG1*, NM_004130.3; PAS, periodic acid–Schiff; Pt, patient; UK, United Kingdom; y, years.

### Western blot analysis of glycogenin-1 and glycogenin-2

Western blot was performed on protein extracted from fresh-frozen skeletal muscle, cardiac muscle, and liver tissue. To identify glycogenin, which is located inside the large glycogen molecules, digestion with α-amylase is necessary to degrade glycogen and obtain free glycogenin protein for gel electrophoresis. Fifty U/mL human α-amylase (Sigma-Aldrich) in a total volume of 25 μL phosphate-buffered saline pH 6.5 was added to approximately 1 to 2 mg muscle tissue cut into sections in a cryostat and incubated at 37°C for 1 hour, before proceeding to protein extraction and sodium dodecyl sulfate-polyacrylamide gel electrophoresis. The transfer was performed using the iBlot Dry Blotting System (Life Technologies). The membrane was blocked for 1 hour at room temperature with SuperBlock blocking buffer (Thermo Fisher Scientific) followed by overnight incubation at 4°C with the primary antibodies. Glycogenins were detected with primary antiglycogenin-1 antibody M07 clone 3B5 (Abnova) at a dilution of 1:500 and primary antiglycogenin-2 antibody (HPA005495; Atlas Antibodies) at a dilution of 1:1000. For detection, we used horseradish peroxidase–conjugated secondary antibodies (Thermo Fisher Scientific, 1:1000). SuperSignal West Dura Extended Duration Substrate or SuperSignal West Femto Maximum Sensitivity Substrate (Thermo Fisher Scientific) was applied and an enhanced chemiluminescent detection Western blotting system (Fujifilm LAS-4000 system) was used for visualization.

### Morphological and immunohistochemical analysis

Histochemical analyses of skeletal and cardiac muscle were performed on fresh-frozen tissue specimens and formalin-fixed, paraffin-embedded liver specimens. Eight-μm-thick cryostat sections were fixed in acetone for 10 minutes and washed in TRIS-buffered saline-Tween for 10 minutes; 5-μm-thick paraffin sections were used without fixation. The specimens were stained with periodic acid–Schiff (PAS) reagent using a standard protocol to determine the presence of glycogen (17). Immunohistochemical detection of free glycogenin-1 (M07 clone 3B5; Abnova, 1:500) and glycogenin-2 (HPA005495; Atlas Antibodies, 1:100) was performed using a Dako Autostainer and using the Dako EnVision FLEX High pH kit according to the manufacturer’s standard protocol. Primary antibodies were applied for 1 hour. The Dako Liquid DAB+ Substrate Chromogen System was used to visualize positive staining material. For electron microscopy, small tissue specimens were directly fixed in glutaraldehyde in phosphate buffer, postfixed in osmium tetroxide, and embedded in resin. The contrast of ultrathin sections was enhanced by uranyl acetate and lead citrate.

### Mass spectrometry

To identify glycogenin-2 protein in polyacrylamide gels, liquid chromatography-tandem mass spectrometry (MS) analysis was performed. Proteomic analyses were performed at the Proteomics Core Facility at the Sahlgrenska Academy, University of Gothenburg, according to standard protocols. Gel pieces corresponding to functional glycogenin-2, identified by Western blot analysis, were destained with 25 mM ammonium bicarbonate in 50% acetonitrile, in-gel digested by addition of 10 ng/μL trypsin (Pierce MS grade, Thermo Fisher Scientific) in 50 mM ammonium bicarbonate, and incubated overnight at 37°C. Samples were analyzed on an Orbitrap Fusion Tribrid mass spectrometer interfaced with an Easy nLC 1200 liquid chromatography system (Thermo Fisher Scientific). Peptides were trapped on an Acclaim Pepmap 100 C18 trap column (100 μm × 2 cm, particle size 5 μm; Thermo Fischer Scientific) and separated on an in-house–packed analytical column (75 μm × 300 mm, particle size 3 μm, Reprosil-Pur C18; Dr. Maisch) using a linear gradient of acetonitrile from 5% to 80% in 0.2% formic acid over 25 minutes at a flow rate of 300 nL/min. Precursor ion mass spectra were acquired at 120 000 resolution and MS/MS analysis was performed in a data-dependent mode by which collision-induced dissociation spectra of the most intense precursor ions were recorded in an ion trap with a collision energy setting of 35 for 3 seconds (“top speed” setting). Charge states 2 to 7 were selected for fragmentation, and dynamic exclusion was set to 30 seconds.

Data analysis was performed using Proteome Discoverer version 1.4 (Thermo Fisher Scientific) against the Human Swissprot Database, March 2017. Mascot 2.5 (Matrix Science) was used as a search engine with a precursor mass tolerance of 5 ppm and a fragment mass tolerance of 500 mmu. Tryptic peptides were accepted with 1 missed cleavage, variable methionine oxidation, and static cysteine propionamide modifications. The detected peptide threshold in the software was set to a significance level of Mascot 95% by searching against a reversed database, and identified proteins were grouped by sharing the same sequences to minimize redundancy.

### Ethical statement

The study complied with the Declaration of Helsinki and was approved by the regional ethical review board in Gothenburg, Sweden. The investigated individuals gave their informed consent.

## Results

### Validation of antibodies

Glycogenin-1 and glycogenin-2 are normally embedded in glycogen particles and are not available for immunodetection by Western blot analysis or by immunohistochemistry. This characteristic of glycogenin-1 and glycogenin-2 can be used to validate the specificity of the antibodies used because functional glycogenin in normal liver and muscle can be detected only after digestion of the glycogen, for example, by using α-amylase.

**Figure 1.**
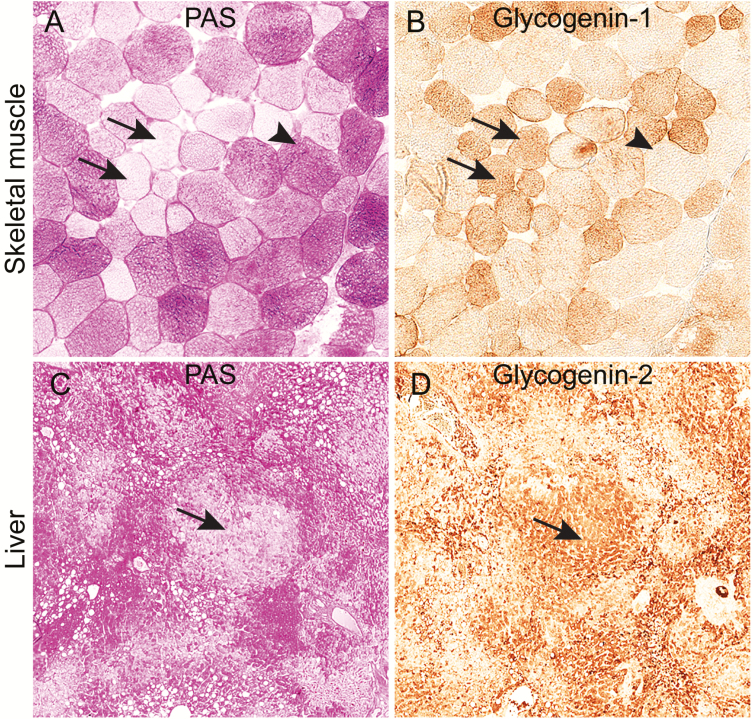
Glycogen storage and immunohistochemical expression of glycogenin in serial sections of skeletal muscle and liver of controls without glycogenin deficiency. A, Analysis of glycogen storage by staining with periodic acid–Schiff (PAS) reagent in a control muscle sample showing some pale muscle fibers (arrows) in which glycogen has been used, and also fibers with normal glycogen content (arrowhead). B, Immunohistochemical staining using an antibody to glycogenin-1. In the fibers depleted of glycogen, the glycogenin-1 molecules, which are normally covered by polysaccharide chains, are exposed and expression of glycogenin-1 is detectable (arrows). Fibers with normal glycogen content do not show glycogenin-1 expression by immunohistochemical staining (arrowhead). C, PAS staining of control liver showing pale regions (arrow) where glycogen has been used and the glucose molecules are digested. D, Immunohistochemical staining using an antibody to glycogenin-2. In the pale regions in the PAS staining, glycogenin is exposed and the expected expression of glycogenin-2 is evident (arrow).

**Figure 2.**
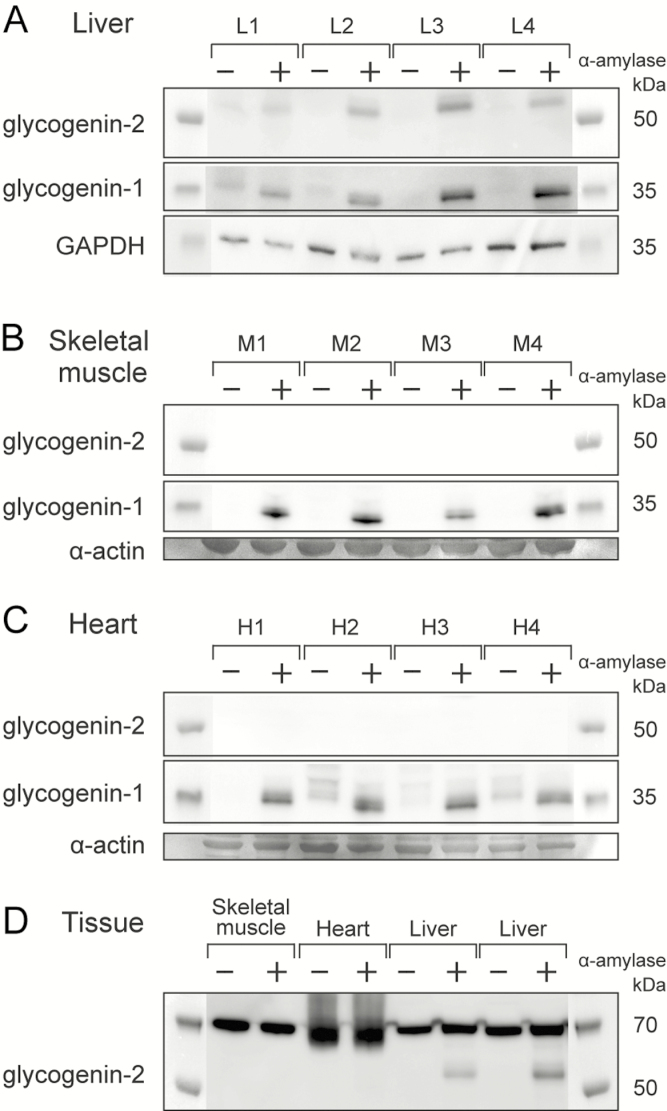
Western blot analysis of glycogenin-1 and glycogenin-2 in control tissues. A, In liver controls (L1-L4), glycogenin-1 and glycogenin-2 both were detectable after α-amylase treatment (+). B, In skeletal muscle (M1-M4), only glycogenin-1, but not glycogenin-2, was detectable after α-amylase treatment (+). C, In heart (H1-H4), glycogenin-1, but not glycogenin-2, was detectable after α-amylase treatment (+) and to some degree, even without any α-amylase treatment (–). The slightly larger size (≈1 kDa) of the glycogenin-1 without α-amylase treatment indicates that there was a small amount of functional, autoglucosylated glycogenin-1 in the heart that was not embedded in glycogen molecules. D, A panel of 3 different tissues (heart, skeletal muscle, and liver). A band corresponding to glycogenin-2 and appearing only after α-amylase treatment is present in the liver only. Glycogenin-2 peptides were identified by mass spectrometry (MS) in the gel in the same region, but only in the liver. All tissues show a band at around 70 kDa that is equally strong with or without α-amylase treatment. No glycogenin-2 peptides were identified by MS in gel pieces cut from this region. Loading controls were α-actin (skeletal muscle and heart panels) from gels stained with Coomassie blue (SimplyBlue SafeStain,Thermo Fisher Scientific). GAPDH indicates glyceraldehyde-3-phosphate dehydrogenase.

For glycogenin-1, we used an antibody (M07 clone 3B5) with high specificity for glycogenin-1 in Western blot analysis (6, 15). To check the specificity of the antibody in immunohistochemistry, we studied muscle specimens from patients without glycogen storage disease in whom the biopsy revealed isolated or groups of muscle fibers depleted of PAS-positive glycogen. Such focal depletion of PAS-positive glycogen, which can be seen in some neuromuscular disorders, is presumably a result of uneven consumption of the glucose stored in glycogen molecules, thereby exposing the glycogenin-1 epitopes in such regions (Fig. 1A and 1B). Western blot analysis included protein extracts both with and without α-amylase digestion to check for specificity for functional glycogenin-1 (Fig. 2A-2C).

For glycogenin-2, the antibody HPA005495 was used. To check the specificity of the antibody in immunohistochemistry, we studied liver specimens from patients without glycogen storage disease but in whom the biopsy had groups of liver cells with no PAS-positive glycogen, thereby revealing the glycogenin-2 epitopes (Fig. 1C and 1D).

To check the specificity of the antibody in Western blot analysis, MS was performed on liver proteins that appeared to bind the antibody but only after α-amylase digestion and those with a molecular weight of approximately 55 kDa. Two peptide sequences that aligned with human glycogenin-2 (UniProtKB, O15488) were identified: RPELGLTLTK and NWSTTDIHK. Intense bands with a molecular mass of 70 kDa and with similar intensity irrespective of whether they were treated with α-amylase (Fig. 2D) did not contain any glycogenin-2 peptides by MS. In all Western blot analyses, we included protein extracts that had and those that had not undergone α-amylase digestion to check for specificity (Fig. 2A-2D).

### Glycogenin-1 and glycogenin-2 are expressed in liver but only glycogenin-1 is expressed in heart and skeletal muscle

After verification of the antiglycogenin-2 antibody and its corresponding bands with MS-based protein assays, we performed Western blot analysis and found expression of functional glycogenin-1 and glycogenin-2 in liver controls (Fig. 2A and 2D). In skeletal muscle and heart controls, only functional glycogenin-1 was detected but not glycogenin-2. In contrast to proteins extracted from liver, MS analysis of cardiac muscle protein bands corresponding to 55 kDa did not reveal any glycogenin-2 peptides.

### Glycogenin-2 is not expressed in skeletal muscle or heart in patients with biallelic *GYG1* mutations

**Figure 3.**
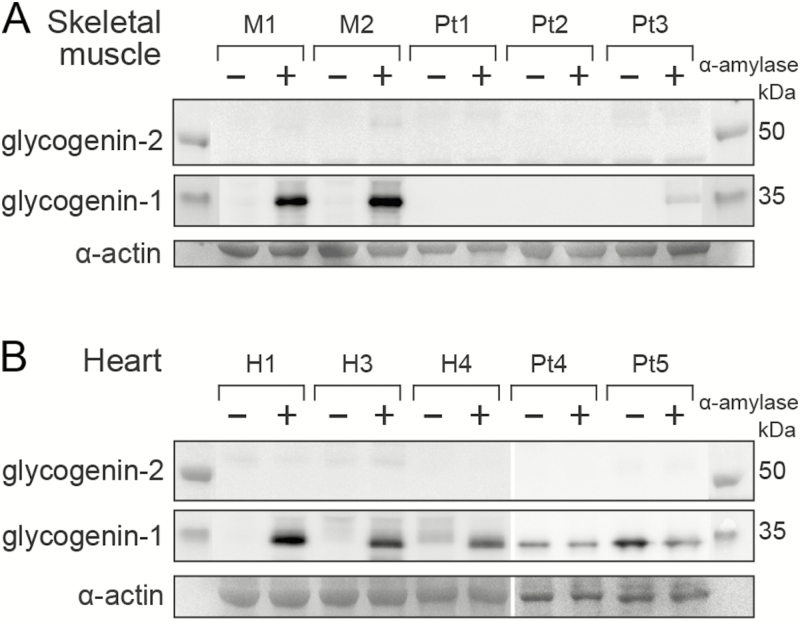
Western blot analysis of glycogenin-1 and glycogenin-2 in patients with biallelic *GYG1* mutations. A, In skeletal muscle, glycogenin-1 was detected only in controls (M1-M2) after α-amylase treatment (+). However, a weak band of normal size was seen after α-amylase treatment in Pt3, indicating a small amount of functional glycogenin-1. Glycogenin-2 was not detected in controls or in patients with *GYG1* mutations (Pt1-Pt3) irrespective of whether there was α-amylase treatment. B, In the heart, glycogenin-1 was detectable after α-amylase treatment (+) in controls (H1, H3, and H4) as well as patients (Pt4 and Pt5). Glycogenin-1 is nonfunctional in the heart in patients with biallelic *GYG1* mutations because it was identified with equally strong bands both with (+) and without (–) α-amylase treatment. It was smaller (≈1 kDa) than the glycogenin-1 identified in 1 of the control hearts without α-amylase treatment (see H4 [–]), indicating that the glycogenin-1 in the patients was not capable of autoglucosylation and therefore not functional. Glycogenin-2 was not detected in controls or patients. The loading controls were α-actin from gels stained with Coomassie blue (SimplyBlue SafeStain,Thermo Fisher Scientific). Pt, patient.

In the 3 patients with myopathy (Pt1, Pt2, and Pt3) only Pt3 showed some residual presence of functional glycogenin-1 because a faint band was detected, but only after α-amylase treatment (Fig. 3A). The residual glycogenin-1 was presumably derived from partially normal splicing. Glycogenin-2 was not detected in any of these patients with myopathy. In the 2 patients with cardiomyopathy, nonfunctional glycogenin-1 was detected in the heart because it was present either with or without α-amylase digestion of the protein extract (Fig. 3B). Glycogenin-2 was not detected in the heart of the patients with *GYG1* mutations and cardiomyopathy.

### Nonfunctional glycogenin-1 protein colocalized with abnormal glycogen storage in the heart

**Figure 4.**
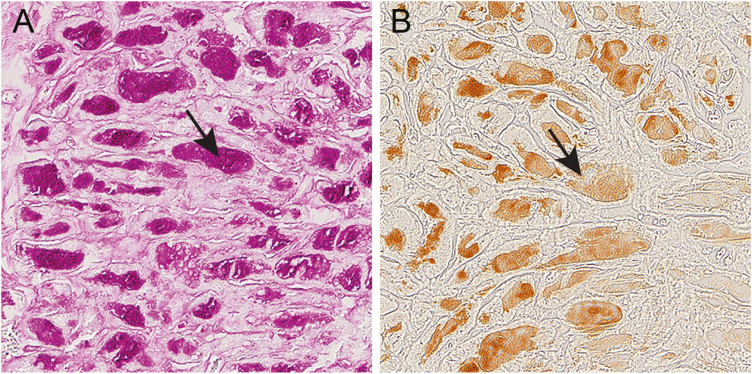
Glycogen and glycogenin-1 in serial sections of the heart in patient 5 with expression of mutated glycogenin-1. A, Endomyocardial biopsy showing abnormal accumulation of periodic acid–Schiff (PAS)-positive material (arrow) in the central part of each cardiomyocyte, corresponding to glycogen and polyglucosan. B, The central inclusions of PAS-positive material stained strongly positive for glycogenin-1 by immunohistochemistry (arrow).

In patients with cardiomyopathy due to *GYG1* mutations, Western blot analysis revealed the presence of nonfunctional glycogenin-1. By immunohistochemistry, this mutated unglucosylated glycogenin-1 was found in the same regions as the central deposits of abnormal glycogen and polyglucosan in the cardiomyocytes (Fig. 4A and 4B).

**Figure 5.**
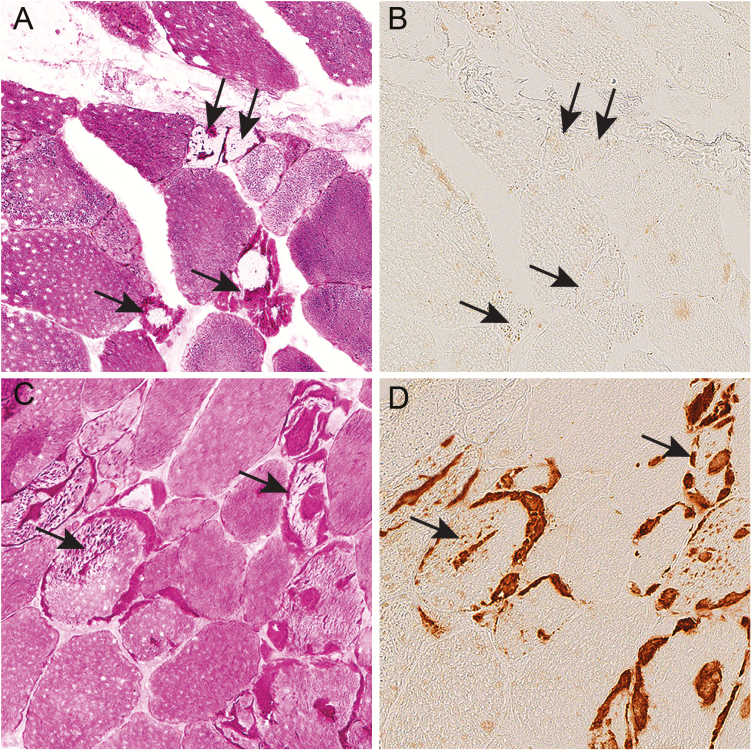
Glycogen and glycogenin-1 in serial sections of skeletal muscle in patients with *GYG1* mutations. A and B, Skeletal muscle biopsy from Pt1 with biallelic start codon *GYG1* mutations, A, showing some fibers with storage of intensely periodic acid–Schiff (PAS)-positive material corresponding to abnormal glycogen and polyglucosan (arrows). B, No glycogenin-1 was present in the inclusions (arrows) or elsewhere. C and D, Skeletal muscle biopsy from patient 3 with biallelic *GYG1* mutations and some residual glycogenin-1 on Western blot analysis. C, Several fibers showed inclusions with storage of intensely PAS-positive material corresponding to abnormal glycogen and polyglucosan (arrows). D, Glycogenin-1 was identified in the inclusions (arrows).

In the patients with *GYG1* mutations and myopathy (Pt1, Pt2, and Pt3), there was accumulation of PAS-positive abnormal glycogen and polyglucosan in several muscle fibers (Fig. 5A and 5C). In Pt1 and Pt2, there was no expression of glycogenin-1 by Western blot analysis (Fig. 3A), which was in accordance with the immunohistochemical findings (Fig. 5B). In Pt3, the residual glycogenin-1 was apparently functional and glucosylated to some extent because it was found in the Western blot analysis only after pretreatment with α-amylase (Fig. 3A). By immunohistochemistry, glycogenin-1 was detected in the regions of storage of abnormal glycogen and polyglucosan (Fig. 5D). In these fibers with abnormal glycogen storage, the glycogenin-1 was probably trapped in the storage material and unable to be normally glucosylated because it was detected by immunohistochemistry.

**Figure 6.**
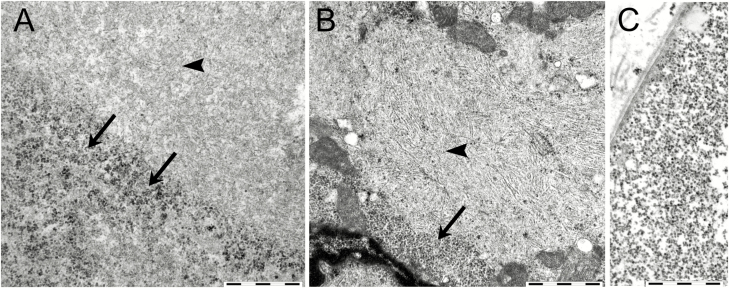
Electron microscopy of glycogen storage in cardiac and skeletal muscle in patients with biallelic *GYG1* mutations. A, In skeletal muscle, the accumulated material consisted of a mixture of granular, apparently normal glycogen (arrows) and fibrillar material (arrowhead). The filamentous material represented large regions of the periodic acid–Schiff–positive storage material and was morphologically compatible with polyglucosan. B, In cardiac muscle, there was a similar mixture of granular material (arrow) and fibrillar material (arrowhead) material. C, Normal muscle glycogen is shown for comparison. Bars correspond to 1 µm.

Electron microscopy of skeletal muscle in the patients with myopathy showed that most fibers contained apparently normal glycogen. In fibers with accumulated PAS-positive material, the glycogen was partly granular but to a great extent the material had a fibrillar structure corresponding to polyglucosan (Fig. 6A). In the heart tissue, both granular and abnormal filamentous glycogen (polyglucosan) was present in the accumulated PAS-positive material (Fig. 6B).

## Discussion

Glycogenin has previously been considered to be essential for de novo glycogen biogenesis by forming an oligosaccharide chain, which acts as a primer on which glycogen synthase and branching enzyme build the large, branched glycogen molecule (2, 18). In this study we investigated the expression of the 2 isoforms of human glycogenin, glycogenin-1 and glycogenin-2, in controls and in patients with glycogenin-1 deficiency. In accordance with previously published results, we detected expression of both glycogenin-1 and glycogenin-2 in liver tissue (3, 4, 19, 20). However, only glycogenin-1 was identified in the skeletal muscle and heart of controls. Glycogenin-2 transcripts have been identified in cardiac muscle in a previous study (4). However, in the Genotype-Tissue Expression database, glycogenin-1 is highly expressed in the heart but glycogenin-2 is not, which is in line with the results from previous protein analyses (20).

In the present study we found no upregulation of expression of glycogenin-2 in patients with glycogenin-1 deficiency, either in heart tissue or in skeletal muscle. The role of glycogenin-2 is not clear. It is mainly expressed in fat tissue (according to the Genotype-Tissue Expression database), but Western blot does not indicate that glycogenin-2 is associated with glycogen in fat tissue because α-amylase treatment does not increase the intensity of the band (20). The importance of glycogenin-2 for liver glycogen metabolism is also unclear because there is a common deletion (affecting 1% of the population [21]), removing the *GYG2* gene, which is located on the X chromosome. Men who are hemizygous for the deletion and therefore completely lack glycogenin-2 have no apparent phenotype, normal blood glucose, a normal glucagon stimulation test, and normal liver glycogen as determined by light and electron microscopy (21).

As shown in this study, glycogenin-1 is also dispensable for glycogen synthesis in muscle and the heart and there is no compensatory upregulation of functional glycogenin-2. These findings are in accordance with the results of studies on glycogenin knockout mice, in which the glycogen content was found to be even higher in glycogenin-deficient mice than in controls (22). Mice have only one glycogenin gene, and no other protein bound covalently to glycogen has been identified, indicating that glycogen synthesis can occur without a protein primer such as glycogenin (22). Synthesis of glycogen without glycogenin being present can also occur in other organisms such as bacteria (23) and yeast (24).

The finding that lack of glycogenin does not induce glycogen depletion indicates that glycogenin may have important functions other than being a primer for glycogen synthesis. It has been demonstrated that glycogenin strongly interacts with glycogen synthase (25), but the functional importance of such interaction is not known. An imbalance between the functional activity of glycogen synthase and branching enzyme has been proposed to be a cause of polyglucosan storage, as seen in equine polysaccharide storage disease, which is caused by a dominant glycogen synthase mutation (26), in mice overexpressing glycogen synthase (27), and in humans with branching enzyme deficiency (28). Whether glycogenin-1 deficiency affects the balance between glycogen synthase and branching enzyme activities in muscle remains to be investigated, and there are many other factors implicated in the formation of polyglucosan such as in Lafora disease, which is another polyglucosan storage disease (29).

Immunohistochemical staining of glycogenin-1 in cardiac muscle revealed accumulation of glycogenin-1 in the regions showing storage of PAS-positive material, which was found by electron microscopy to be composed of a mixture of fibrillar and granular material. This storage of glycogen and polyglucosan thus includes mutated glycogenin-1, probably together with a large number of other proteins that are normally associated with glycogen. The mutated glycogenin-1 is nonfunctional because it lacks the ability to autoglucosylate, but it can probably bind to glycogen synthase, which is 1 of many proteins associated with glycogen (2). An important question is whether expression of mutated glycogenin-1 does in fact cause cardiomyopathy since patients who completely lack glycogenin-1 because of biallelic truncating *GYG1* mutations do not develop cardiomyopathy. In skeletal muscle, glycogenin-1 is therefore not present in the storage material of most patients, as demonstrated in Pt1 in our study.

In conclusion, we have found that glycogenin-1 is dispensable for glycogen synthesis in humans, which has also been shown in mice. This finding challenges the concept of glycogenin being an obligate primer for de novo glycogen synthesis. Glycogenin-1 deficiency leads to glycogen storage and polyglucosan formation, indicating that glycogenin-1 is involved in the regulation of glycogen synthesis in muscle, which needs to be further studied.

## Abbreviations

GYG1: NM_004130.3
MS: mass spectrometry
PAS: periodic acid–Schiff
